# Clinical Epidemiology, Risk Factors, and Control Strategies of *Klebsiella pneumoniae* Infection

**DOI:** 10.3389/fmicb.2021.750662

**Published:** 2021-12-22

**Authors:** De Chang, Lokesh Sharma, Charles S. Dela Cruz, Dong Zhang

**Affiliations:** ^1^Department of Pulmonary and Critical Care Medicine, The Third Medical Center of Chinese PLA General Hospital, Beijing, China; ^2^College of Pulmonary and Critical Care Medicine, Chinese PLA General Hospital, Beijing, China; ^3^Section of Pulmonary and Critical Care and Sleep Medicine, Department of Medicine, Yale University School of Medicine, New Haven, CT, United States; ^4^Department of Oncology, The Second Medical Center of Chinese PLA General Hospital, Beijing, China; ^5^College of Tuberculosis Medicine, Chinese PLA General Hospital, Beijing, China

**Keywords:** *Klebsiella pneumoniae*, clinical epidemiology, risk factors, control strategies, hvKp, CRKP

## Abstract

*Klebsiella* species cause infections at multiple sites, including lung, urinary tract, bloodstream, wound or surgical site, and brain. These infections are more likely to occur in people with preexisting health conditions. *Klebsiella pneumoniae* (*K. pneumoniae*) has emerged as a major pathogen of international concern due to the increasing incidences of hypervirulent and carbapenem-resistant strains. It is imperative to understand risk factors, prevention strategies, and therapeutic avenues to treat multidrug-resistant *Klebsiella* infections. Here, we highlight the epidemiology, risk factors, and control strategies against *K. pneumoniae* infections to highlight the grave risk posed by this pathogen and currently available options to treat *Klebsiella*-associated diseases.

## Introduction

*Klebsiella pneumoniae* is a Gram-negative, encapsulated, non-motile, facultatively anaerobic bacteria (Ray and Ryan, 2004). It was first isolated from the airways of a patient dying of pneumonia by Edwin Klebs in 1875 and later described by Carl Friedländer in 1882, leading it to be called Friedlander’s bacillus for some time (Köhler and Mochmann, 1987). *Klebsiella* species include *Klebsiella ozaenae*, *Klebsiella rhinoscleroma*, and *K. pneumoniae*, the last of which is an important opportunistic and iatrogenic infectious pathogen with major clinical implications (Bagley, 1985). In humans, *Klebsiella* often colonizes the nasal and digestive tract without causing any symptomatic disease. However, the colonization can turn into an infection when the host immunity fails to control the pathogen growth, examples of which include patients with diabetes, on glucocorticoid therapy, and those who have received organ transplantation. This mini-review will discuss critical aspects of *K. pneumoniae* biology related to its pathogenesis and control strategies.

## Clinical Epidemiology

### Colonization and Infection

*Klebsiella* species are present abundantly in nature and commonly found in soil, water, and other surfaces (Martin and Bachman, 2018). In humans, *K. pneumoniae* often colonizes at various mucosal surfaces, including the upper respiratory tract and the gut, where colonization rates vary widely among individuals based on their habitat and exposures (Bagley, 1985; Podschun et al., 2001; Martin and Bachman, 2018). Recent studies showed that the prevalence of *Klebsiella* colonization ranges from 18.8 to 87.7% in Asia and 5 to 35% in Western countries (Lin et al., 2012; Russo and Marr, 2019). In the non-hospital settings, the carrier rate of *Klebsiella* in fecal samples ranges from 5 to 38%, while the carrier rates in the nasopharynx range from 1 to 6% (Fuxench-López and Ramírez-Ronda, 1978; Podschun and Ullmann, 1998). The presentation of *Klebsiella* involving the skin is rare, which is considered transient rather than persistent (Kloos and Musselwhite, 1975). In hospitalized patients, colonization rates in the nasopharynx rise to 19%, while it can be as high as 77% in the gastrointestinal tract (Pollack et al., 1972; Podschun and Ullmann, 1998). Due to its extensive presence in humans, gastrointestinal colonization serves as a major reservoir for transmission and infection to other sites (Martin et al., 2016; Dorman and Short, 2017). The experimental evidence of gastrointestinal sources of infection came from a study by Selden et al. (1971), which demonstrated that *K. pneumoniae* infections often show similar serotypes as colonizing bacteria in the intestinal tract. More recent studies have confirmed the relationship between colonizing strains of *K. pneumoniae* and strains obtained from the infection sites (Martin et al., 2016; Dorman and Short, 2017). A longitudinal study confirmed these studies where investigators determined the colonization of *Klebsiella* in the gastrointestinal tract of a cohort of 1765 patients and followed this cohort for 3 months to assess the respiratory tract, urinary tract, or blood infections. The results demonstrated that 21 of 406 patients (5.2%) had colonization developed a subsequent infection compared to only 1.3% of the subjects without colonization. Genetic sequencing revealed that most of the *Klebsiella* infections originated from the colonization sites in the same patient.

Similarly, Gorrie et al. (2017) analyzed the relationship between colonization and susceptibility to infection by *K. pneumoniae* in 498 ICU patients. They found that 16% of the patients colonized with *K. pneumoniae* were found to be infected, compared to only 3% in the non-carriers (Gorrie et al., 2017). Whole-genome sequencing revealed that the patients were infected with the same strain they carried in the form of colonization. From a genomic perspective, these studies demonstrated that gastrointestinal microbiota is a prominent source of nosocomial *K. pneumoniae* infections, 80% of which are caused by self-colonizing strains. The transition from colonization to infection is primarily due to the impairment of host defense contributed by underlying diseases or immunomodulatory therapies (Tsay et al., 2002; Meatherall et al., 2009). To support this notion, a study by Lee et al. (1994) observed 101 patients diagnosed with *Klebsiella* bacteremia and found that 36% of patients had diabetes, and 26% had malignant tumors (Tsai et al., 2010).

Discriminating between colonization and infection has always been a puzzle for clinicians and researchers, making it difficult for subsequent intervention strategies. However, several factors can be considered to distinguish colonization from infection. These factors include:

1. Detection of *Klebsiella* in the bloodstream indicates an active infection. Circulating blood remains sterile, unlike the respiratory, urinary, and digestive tracts, which harbor colonizing *K. pneumoniae*.
2. The patient’s symptoms, clinical signs, laboratory examination, and imaging data should be employed to differentiate colonization from infection. For example, respiratory infection with *Klebsiella* is diagnosed if the patient has a fever, cough, sputum production, high WBC, and imaging evidence of pneumonia in the lung.
3. When patients have underlying diseases such as COPD, diabetes, heart disease, organ transplantation, or a recent history of steroid use or antimicrobial drugs, the infection of *Klebsiella* should be considered when positive cultures are obtained. In summary, infections by *K. pneumoniae* often derive from the colonizing bacteria within the host, and both clinical and bacteriological factors should be considered to distinguish active infection from colonization.

### Hypervirulent *K. pneumoniae*

Hypervirulent *K. pneumoniae* (hvKp) indicates a *K. pneumoniae* strain that can cause infections in relatively healthy subjects, often in community settings. The infection is often manifested in multiple organs (Struve et al., 2015). HvKp was first reported in 1986 in Taiwan, which caused pneumonia complicated with liver abscess, meningitis, and endophthalmitis (Liu et al., 1986). This strain causing liver abscess was also known as hypermucoviscous *K. pneumoniae* due to its hypermucoviscous phenotype (string test >5 mm), as described by Fang et al. (2004). Unique sequences on the plasmid can distinguish hvKp from classic *K. pneumoniae* strains (Russo et al., 2018). The high virulence of these strains is recapitulated in animal models as demonstrated by a 50% lethal dose (LD50) being as low as 10^3^ colony forming units (Nassif and Sansonetti, 1986). The hypermucoviscous phenotype is contributed by a unique plasmid in some of these strains (Nassif et al., 1989; Nassrf et al., 1989). In this regard, the first strain isolated by Friedlander in 1882 was considered as hvKp because it was highly pathogenic and could infect multiple sites in the body (Russo and Marr, 2019). However, the subsequent studies demonstrated that not all hvKp have hypermucoviscous phenotype, and some common *K. pneumoniae* strains were hypermucoviscous, indicating that hypermucoviscous phenotype can be present in non-hypervirulent strains of *Klebsiella* (Catalán-Nájera et al., 2017; Russo et al., 2018). At present, cases of hvKp have already been reported from Europe, Asia, and the United States (Russo and Marr, 2019).

The first case of hvKp infection in North America was a 38-year-old African American man with headache and fever diagnosed with a liver abscess caused by hvKp and complicated by endophthalmitis and meningitis (Saccente, 1999; Table 1). In a retrospective study of 56 cases of liver abscess at Elmhurst Hospital in New York City, 36 percent of the patients were caused by hvKp using imaging and culture of blood or liver aspiration (Pastagia and Arumugam, 2008). Subsequently, more and more hvKp infections were reported (Pastagia and Arumugam, 2008; Mccabe et al., 2010; Fierer et al., 2011; Vila et al., 2011). Increasing reports of the multiorgan clinical manifestation associated with *K. pneumoniae* such as liver abscess, bacteremia, meningitis, endophthalmitis, and necrotizing fasciitis indicate a high prevalence hvKp, which has become a global disease that requires immediate attention. The earliest clinical clues for hvKp include the presence of liver abscess and bacteremia in patients with a positive culture. Due to high lethality, early diagnosis and intervention play a key role in limiting the disease severity and death due to hvKp.

**Table 1:** The first case of hypervirulent *K. pneumoniae* (hvKp) in each continent.

Continent	Country	Year	Disease	Culture origin	Reference
Asia	Taiwan, China	1986	Liver abscess; septic endophthalmitis	Blood	Liu et al., 1986
North America	Arkansas, United States	1999	Liver abscess, endophthalmitis, and meningitis	CSF	Saccente, 1999
South America	Mendoza, Argentina	2011	Liver abscess	Blood and abscess	Vila et al., 2011
Europe	Granada, Spain	1999	Liver abscess	Blood	Cobo et al., 1999
Africa	Johannesburg, South Africa	2007	Liver abscess and meningitis	Blood	Meents and Boyles, 2010
Australia	Sydney, Australia	1997	Liver abscess metastatic septic endophthalmitis	Urine and blood	Lindstrom et al., 1997
Antarctica	None				

### Carbapenem-Resistant *K. pneumoniae*

Pathogens employ multiple mechanisms to develop antibiotic resistance, including the production of beta-lactamase, loss of susceptible outer membrane proteins, change of target, biofilm formation, efflux pump, and integron (Thomson and Bonomo, 2005). Exposure to antibiotics at sublethal concentrations may lead to the development of resistance among exposed pathogens. In addition to the clinical misuse of antimicrobial agents, the human population is often exposed to a wide range of non-iatrogenic antibacterial drugs in daily life, including antibiotic exposures to the livestock in the meat industry, which leads to increased drug resistance in pathogens. Increased use of antibiotics in both clinical and non-clinical settings is associated with an increased number of clinically isolated drug-resistant strains, including carbapenem-resistant *K. pneumoniae* (CRKP; Fair and Tor, 2014; Li and Webster, 2018).

In recent years, the extensive use of carbapenems in clinical practice increased carbapenem resistance in *K. pneumoniae*. Since its emergence in the 1990s, it has gradually become prevalent worldwide with high mortality (Table 2). The presence of carbapenem-resistance in the infecting pathogens is an independent risk factor of mortality among patients with nosocomial infection. There are several mechanisms responsible for carbapenem resistance among *K. pneumoniae*. The primary mechanism is the production of a carbapenemase classified into the Ambler class A (*K. pneumoniae* carbapenemase, KPC), B (the metalloenzymes NDM, VIM, and IMP), and D (oxacillin enzyme, OXA; Spagnolo et al., 2014; Logan and Weinstein, 2017).

**Table 2:** The first report of representative carbapenem-resistant *K. pneumoniae* (CRKP).

Strain	Year	Country	Ambler structural class	Molecular epidemiology	Reference
KPC-1-KP (1534)	1996	United States	A	Tn4401	Yigit et al., 2001
NDM-1-KP (05-506)	2008	India	B	N plasmids	Yong et al., 2009
OXA-48-KP (11978)	2001	Turkey	D	Tn1999	Poirel et al., 2004

The coding gene of KPC is *blaKPC*, which can be transferred by the plasmid among *K. pneumoniae* strains through Tn3-based transposon Tn4401 (Spagnolo et al., 2014; Logan and Weinstein, 2017). The *blaKPC* gene can also be transmitted to other bacteria like *Enterobacter* and *Pseudomonas aeruginosa* (Virgincar et al., 2011). The blaKPC-K*. pneumoniae* strains have been isolated across the globe in recent years, indicating their global presence. The first reported blaKPC-K*. pneumoniae* strain was isolated in a hospital located in North Carolina in 1996 and submitted to the Centers for Disease Control and Prevention (CDC) through the intensive care antimicrobial resistance epidemiology program (ICARE; Yigit et al., 2001). Naas et al. (2005) reported the first French case of CRKP in 2005, an 80-year-old man with prostate cancer and metastasis. These cases demonstrated the transmission of *blaKPC-K. pneumoniae* among different continents.

New Delhi Metallo-beta-lactamase 1 (NDM-1) *K. pneumoniae* is a newly emerged highly resistant bacteria that can produce NDM-1 capable of breaking down beta-lactam antibiotics, belonging to Ambler class B carbapenemase (Kumarasamy et al., 2010). The bacteria with NDM-1 genes were considered “superbugs” due to their lack of susceptibility to almost all available antibiotics (Moellering, 2010). The resistance gene was located not only in the bacterial genome but also in plasmids. Thus, the strains that are initially susceptible to antibiotics may quickly become resistant through horizontal gene transfer. The first NDM-1 *K. pneumoniae* was reported in a 59-year-old Indian male with type II diabetes and multiple strokes. The patient was a resident of Sweden with multiple visits to India (Yong et al., 2009). The strain was isolated from the urine sample on January 9, 2008, when the patient had no apparent urinary tract infection symptoms. To date, NDM-1 *K. pneumoniae* has also been reported across the world including in China, Australia, the United States, Canada, Europe, and Africa (Dhainaut et al., 2005; Burgner et al., 2006; Vered et al., 2014; Hammer et al., 2018).

OXA-48 is an Ambler class D carbapenemase encoded by the *blaOXA*-48 gene, which has been reported increasingly among the *Enterobacteriaceae* family of bacteria in recent years. Although this enzyme has relatively weak β-lactamase activity, it can hydrolyze penicillin and cannot be inhibited by β-lactamase inhibitors. It can spread widely among bacteria through the Tn199-based plasmid containing the *blaOXA*-48 gene flanked with the IS1999 sequence (Pfeifer et al., 2012). OXA-48 was first identified in Turkey in a *K. pneumoniae* in 2001, which was resistant to almost all beta-lactams, including penicillin, cephalosporins, monocyclic lactams, and carbapenems (Poirel et al., 2004). Further analysis revealed that SHV-2a, TEM-1, and OXA-47 were expressed, and antibiotic susceptible extracellular membrane proteins were absent, leading to their resistance to various antibiotics. In subsequent years, outbreaks of the OXA-48 strains occurred in various regions of Turkey. In addition to Turkey, OXA-48 has been reported in other European countries, America, Asia, Oceania, and Africa (Nordmann et al., 2011; Espedido et al., 2013; Van Duin and Doi, 2017). Notably, the incidence of the OXA-48-expressing strains may be underestimated because it is difficult to recognize the presence of OXA-48-like enzymes due to their low levels of carbapenem resistance.

It has been reported that overexpression of efflux pumps, decreased permeability of outer membrane proteins, and production of the β-lactamase enzymes are also important reasons for CRKP (Zhang et al., 2014; Hamzaoui et al., 2018). Carbapenems are described as our last line of defense against drug-resistant bacteria. Most frightening of all, CRKP can pass on its resistance to other bacteria through horizontal gene transfer by various methods, including bacterial conjugation, leading to drug resistance. In particular, the emergence of highly virulent CRKP strains named ST11 CR-HvKp, which are highly virulent and resistant to carbapenems, poses a major challenge to our ability to control *Klebsiella* infections (Yao et al., 2018). The ST11 CR-HvKp strain infects the lungs and causes pneumonia, and invades the blood and other organs, potentially resulting in incurable and fatal infections in relatively healthy individuals.

## Risk Factors

Susceptibility to *K. pneumoniae* infection is determined by pathogen variables (such as virulence factors and antibiotics resistance), host intrinsic (such as genetics, age, and immune status), and extrinsic factors (such as antibiotic use, environmental exposure, nutrition, and alcoholism), among others (Jong et al., 1995; Paczosa and Mecsas, 2016; Martin and Bachman, 2018; Liu and Guo, 2019).

Various virulence factors have been demonstrated to aid in the infectivity of *K. pneumoniae*. These virulence factors include capsule, lipopolysaccharide, adhesin, and siderophores, which are more frequent in CRKP/hvKp, leading to various immune responses and related phenotypes observed in the hvKp strains (Paczosa and Mecsas, 2016; Gomez-Simmonds and Uhlemann, 2017; Martin and Bachman, 2018).

The host provides nutrition and shelter to the bacteria. At the same time, the effective immune system controls bacterial replication to prevent infection. Host factors associated with increased host susceptibility include heredity, age, and underlying diseases (Dhainaut et al., 2005; Burgner et al., 2006; Hammer et al., 2018). Vered et al. (2014) studied the host’s susceptibility to *K. pneumoniae* using quantitative trait locus (QTL) mapping and collaborative cross (CC) mice. They identified host candidate genes for *K. pneumoniae* infection, including *Ctnnal1*, *Actl7a*, *Actl7b*, and *Bag4* (Vered et al., 2014). Newborns, especially those born prematurely or in intensive care units, are at increased risk due to underdeveloped immune systems and immature mucosal barriers of the gastrointestinal tract. On the other end of the spectrum, the elderly subjects have the highest risk of death from *K. pneumoniae*. It is estimated that a mortality rate of 30% in older subjects following hospitalization due to *K. pneumoniae* infection is mainly caused by aspiration of oropharyngeal flora (Afroza, 2006; Collado et al., 2015). Studies on patients whose mean ages were more than 60 years showed that 17.2% of all CAPs and 6.5–11.6% of all HAPs were caused by *K. pneumoniae* (Teramoto et al., 2015). Moreover, diabetes, malignancy, liver and gallbladder disease, chronic obstructive pulmonary disease, renal failure, and nutritional status are additional risk factors, often associated with aging, toward increased susceptibility (Paczosa and Mecsas, 2016).

External factors include antibiotics and glucocorticoids, chemotherapy, transplantation, dialysis, hospital and ICU stays, personal habits, invasive medical procedures such as an endoscope, hypodermic injection, percutaneous surgery, and implantation (Tietjen et al., 2003; Li et al., 2019). Many of these procedures can either contribute to the disruption of the mucosal barrier at the colonization site and allow the pathogen to escape the colonizing site to establish infections, or they directly give the pathogens access to body sites such as intubation.

## Control Strategies

### Source Control

Identifying and eliminating the source of *K. pneumoniae* can effectively avoid the infection by these bacteria. However, major challenges remain in the identification and elimination of the source of infection. Specimen culture is still the primary method for most hospitals to screen for presence of *K. pneumoniae* (Podschun and Ullmann, 1998; Lolans et al., 2010). Some molecular approaches have been reported to identify chromosomal genes such as *blaSHV*, *blaLEN*, *blaOKP*, and their side-chain genes (*deoR*) by multiplex polymerase chain reaction (Fonseca et al., 2017). It is critical to control the sources of hvKp and CRKP. The Carb NP test and molecular identification have been used to screen *Enterobacteriaceae* producing carbohydrase, especially for CRKP in asymptomatic carriers (Dortet et al., 2014).

**Figure 1:**
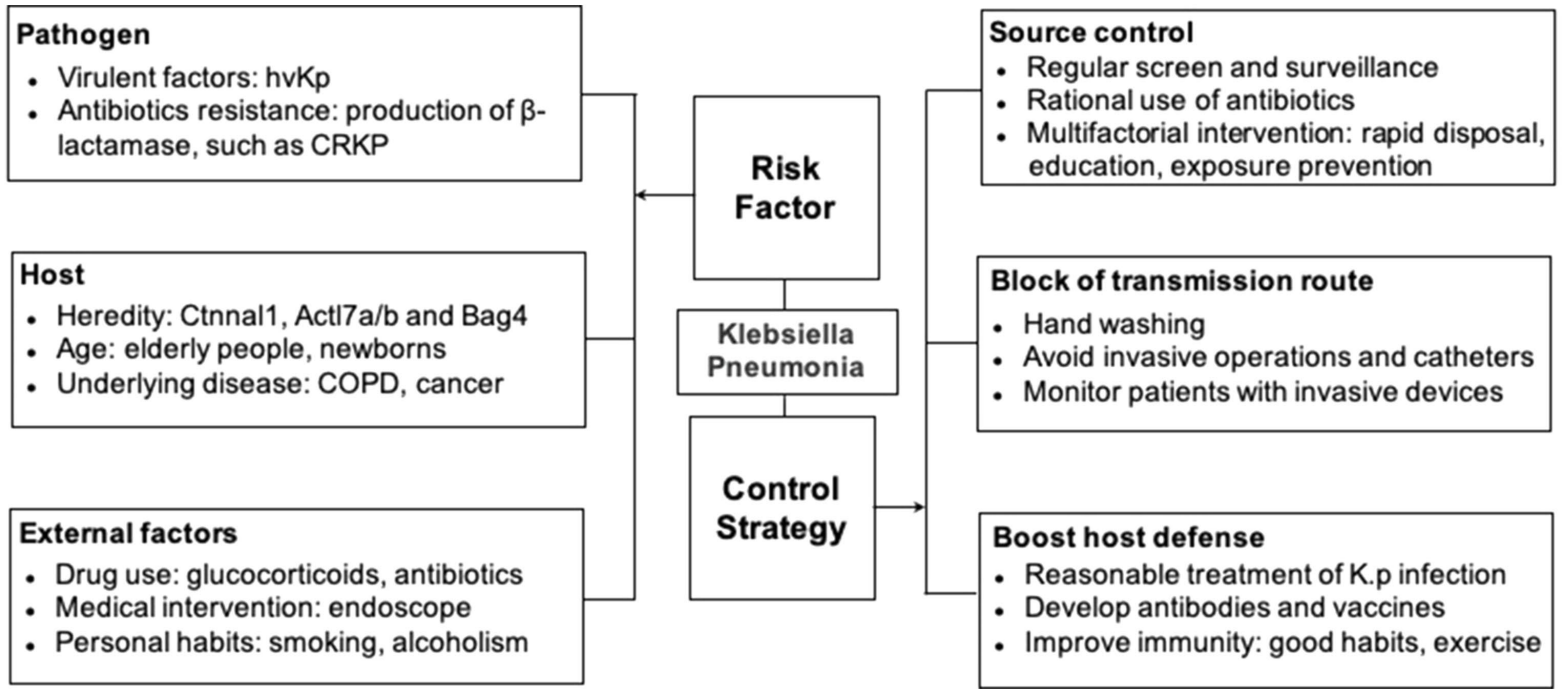
Risk factors and prevention strategies.

Extensive screening, identification, education, and multifactorial intervention are essential to control the spread of *K. pneumoniae* from the source (Spagnolo et al., 2014; Figure 1). Exposure prevention should be conducted by timely identifying the infected individuals and following standard precautions including the use of personal protective equipment such as gowns, gloves, and masks. Contact tracing should be employed whenever possible to minimize further exposure to uninfected subjects, both in hospital and community settings. Education on hand hygiene is also vital for medical workers (Akova et al., 2012; Shobowale et al., 2016; Kang et al., 2019).

The use of antibiotics should be regulated strictly under the guidelines and principles, especially for initial empirical treatment (Guven and Uzun, 2003; Kollef and Micek, 2005). The treatment of hvKp and CRKP should follow their specific treatment guidelines (Calfee, 2017). Rational and standardized use of antibiotics, which includes particular indications, adequate dose, sufficient duration of treatment, cautious change of antibiotics, and other interventions such as surgical drainage and removal of implants, whenever possible, should be followed (Weichman et al., 2013). Moreover, abuse of antibiotics should be minimized, which occurs in the medical setting and during livestock breeding (Levy and Marshall, 2004). Controlling antibiotic use in non-human environments can contribute to limiting the source of CRKP.

### Prevention of Transmission/Block of the Transmission Route

As mentioned above, hand washing is considered the most critical and practical measure to prevent the spread of pathogens (Pittet, 2001; WHO, 2009). Microbes found on healthcare workers include *Klebsiella*, *Staphylococcus aureus*, *Clostridium difficile*, and other Gram-negative bacteria (Haque et al., 2018). Healthcare workers’ hands can be contaminated by direct contact with patients or touching contaminated surfaces in the hospital setting.

The use of invasive surgeries and indwelling devices, including central venous catheters, endotracheal catheters, should be avoided and limited whenever possible or used for a minimum possible duration (O’Grady et al., 2002; Trautner and Darouiche, 2004).

For patients with indwelling devices, specimens should be screened for the presence of bacterial pathogens from relevant sites, such as skin, urine, sputum, and wound secretions (Baron et al., 2013). The transmission among patients can be blocked by monitoring the compliance of contact precautions and specimen culture results, which should be communicated with the healthcare workers to make appropriate and timely decisions in situations, where unexpected transmission is observed. Contact precautions should be taken for the CRKP/HvKp, especially in a population with a high risk of transmission or those who come in contact with infected individuals (Magiorakos et al., 2017). High-risk patients should be screened at admission and periodically afterward for CRKP/HvKp during their hospital stay. Standard contact precautions should be taken for patients with a low risk of transmission and people who have an epidemiological connection with unrecognized or infected or colonized CRKP/HvKp patients. Pre-emptive contact precautions can be taken, while admission monitoring test results are pending, especially for patients admitted from other hospitals known to have HvKp/CRE (Spagnolo et al., 2014).

### Host Defense and Protection of the Susceptible Population

*Klebsiella pneumoniae* is covered with polysaccharides, including the capsule and lipopolysaccharides, which are ideal candidate antigens that can be targeted by vaccine or host immunity (Podschun and Ullmann, 1998). The antibodies and vaccines against capsular polysaccharides are being developed but facing difficulties due to a total of 77 different capsules and nine different kinds of LPS serotypes present in the *K. pneumoniae* (Ahmad et al., 2012; Diago-Navarro et al., 2017, 2018; Hegerle et al., 2018). It is crucial to identify highly conserved antigens of *K. pneumoniae* strains to develop antibodies or vaccines with comprehensive coverage and extensive protection across strains (Lundberg et al., 2013).

Other methods to improve immunity include maintaining a healthy lifestyle, including regular exercise, ensuring enough sleep, smoking cessation, healthy diets, including fruits and vegetables (Monye and Adelowo, 2020). Due to high resistance against a wide range of antibiotics, alternative therapies should be explored. Bacteriophage therapy is a new therapeutic method that can replace or supplement traditional antibiotics (Matsuzaki et al., 2005; Hung et al., 2011; Gu et al., 2012). Phase therapy has demonstrated many advantages, including its specificity, high efficiency, fewer side effects, and lower cost.

The antibiotic treatment regimen against *K. pneumoniae* is usually determined by bacterial culture and subsequent antibiotic sensitivity tests (Giuliano et al., 2019). For patients with gangrene, abscess, and empyema, surgery or interventional treatment should be performed. For community-acquired pneumonia, the empirical antimicrobial therapy should provide adequate coverage for potential Gram-negative pathogens. Third-generation cephalosporin or quinolones should be used for at least 2 weeks, either alone or with aminoglycosides (Metlay et al., 2019). For patients with hospital-acquired *K. pneumonia*, appropriate antibiotic regimens should be used either alone or in combination for at least 14 days, including imipenem, third-generation cephalosporin, quinolones, or aminoglycosides (Qureshi, 2015). Quinolones could be administered intravenously if patients respond rapidly. Carbapenems should be considered the treatment of choice against Extended-Spectrum Beta-Lactamase (ESBL)-producer stains, colistin, tigecycline, and intravenous fosfomycin should be chosen for the strains producing carbapenems (Yu and Chuang, 2015).

In summary, *K. pneumoniae* is an important pathogen for respiratory tract infections, often leading to severe pneumonia and multiorgan infections. It can also cause urinary tract infection, meningitis, sepsis, and biliary tract infection in hospitalized patients, entering the human body through the contaminated respirator, atomizer, or catheters in addition to the self-contamination from the colonized bacteria. In recent years, the emergence of HvKp and CRKP strains has become a major challenge in clinical practice. Further studies on virulence and resistance determinants, genetic lineage information, dissemination mechanisms, effective diagnosis methods, potential antibacterial targets, and prevention measures are needed to help reduce the occurrence and spread of the *K. pneumoniae* infection and associated morbidity and mortality.

## Author Contributions

DC wrote the first draft of the manuscript. LS, CD, and DZ wrote sections of the manuscript. All authors contributed to the article and approved the submitted version.

## Funding

This study was supported by funding from The National Key Research and Development Program of China (SQ2021YFC2300197), Beijing Nova Program Interdisciplinary Cooperation Project (DC; No. Z191100001119021), and the Chinese PLA General Hospital Youth Project (DC; No. QNF19074).

## Conflict of Interest

The authors declare that the research was conducted in the absence of any commercial or financial relationships that could be construed as a potential conflict of interest.

## Publisher’s Note

All claims expressed in this article are solely those of the authors and do not necessarily represent those of their affiliated organizations, or those of the publisher, the editors and the reviewers. Any product that may be evaluated in this article, or claim that may be made by its manufacturer, is not guaranteed or endorsed by the publisher.
